# *Vibrio* Phage VMJ710 Can Prevent and Treat Disease Caused by Pathogenic MDR *V. cholerae* O1 in an Infant Mouse Model

**DOI:** 10.3390/antibiotics12061046

**Published:** 2023-06-14

**Authors:** Naveen Chaudhary, Balvinder Mohan, Harpreet Kaur, Vinay Modgil, Vishal Kant, Alka Bhatia, Neelam Taneja

**Affiliations:** 1 Department of Medical Microbiology, Postgraduate Institute of Medical Education and Research, Chandigarh 160012, India; 2 Department of Experimental Medicine and Biotechnology, Postgraduate Institute of Medical Education and Research, Chandigarh 160012, India

**Keywords:** phage, genome, cholera, antibiotic-resistance, mice

## Abstract

Cholera, a disease of antiquity, is still festering in developing countries that lack safe drinking water and sewage disposal. *Vibrio cholerae*, the causative agent of cholera, has developed multi-drug resistance to many antimicrobial agents. In aquatic habitats, phages are known to influence the occurrence and dispersion of pathogenic *V. cholerae*. We isolated *Vibrio* phage VMJ710 from a community sewage water sample of Manimajra, Chandigarh, in 2015 during an outbreak of cholera. It lysed 46% of multidrug-resistant *V. cholerae* O1 strains. It had significantly reduced the bacterial density within the first 4–6 h of treatment at the three multiplicity of infection (MOI 0.01, 0.1, and 1.0) values used. No bacterial resistance was observed against phage VMJ710 for 20 h in the time–kill assay. It is nearest to an ICP1 phage, i.e., *Vibrio* phage ICP1_2012 (MH310936.1), belonging to the class *Caudoviricetes*. ICP1 phages have been the dominant bacteriophages found in cholera patients’ stools since 2001. Comparative genome analysis of phage VMJ710 and related phages indicated a high level of genetic conservation. The phage was stable over a wide range of temperatures and pH, which will be an advantage for applications in different environmental settings. The phage VMJ710 showed a reduction in biofilm mass growth, bacterial dispersal, and a clear disruption of bacterial biofilm structure. We further tested the phage VMJ710 for its potential therapeutic and prophylactic properties using infant BALB/c mice. Bacterial counts were reduced significantly when phages were administered before and after the challenge of orogastric inoculation with *V. cholerae* serotype O1. A comprehensive whole genome study revealed no indication of lysogenic genes, genes associated with possible virulence factors, or antibiotic resistance. Based on all these properties, phage VMJ710 can be a suitable candidate for oral phage administration and could be a viable method of combatting cholera infection caused by MDR *V. cholerae* pathogenic strains.

## 1. Introduction

Cholera causes around 1.4–4.3 million cases and over 21,000–143,000 deaths each year [1]. The disease is endemic and causes outbreaks in several parts of Southeast Asia and Africa. Seven cholera pandemics have been reported till now. Two serogroups, O1 and O139, of *V. cholerae* are mainly responsible for cholera. The serogroup O1 is further divided into two biotypes, El Tor and classical, each of which has Ogawa and Inaba serotypes. The main pathogenesis and virulence of *V. cholerae* is due to the production of cholera toxin (CT) encoded by a bacteriophage harbored by the pathogen [2]. The cornerstone of cholera treatment is fluid and electrolyte replacement therapy. Antibiotics are not compulsory for a successful treatment, but are used as an adjunct therapy. Antibiotics decrease the duration of disease, reduce the volume of diarrhea, and the duration of shedding of the infective organism in stools. Currently, doxycycline, ciprofloxacin, and azithromycin are effectively used for the treatment of cholera [1,2]. However, due to indiscriminate use, multi-drug resistant (MDR) *V. cholerae* have emerged, and the strains are showing resistance not only to first-line agents such as ampicillin, cotrimoxazole, nalidixic acid, and tetracycline, but also to fluoroquinolones such as ciprofloxacin, third-generation cephalosporins, and azithromycin [3]. Resistance has also emerged against ceftriaxone, and NDM-1 (New Delhi metallo-beta lactamase) gene-encoding carbapenem resistance has been reported in India [4,5]. Mass antibiotic prophylaxis is not recommended by the World Health Organization (WHO) as it carries the risk of the development and spread of antimicrobial resistance (AMR). WHO emphasizes that antibiotic treatment should be used only in severely dehydrated patients in conjunction with rehydration therapy and susceptible household contact cases of cholera patients [4]. Cholera is endemic in most parts of India, according to the study conducted by Ali et al. In India, 150 out of 641 districts have reported cholera, and some of those districts, including Chandigarh, have been labeled as “hotspots” for the disease [6]. A total of twenty- nine outbreaks of cholera were reported in and around Chandigarh, an inland area located in north India, from 2002 to 2015 [5]. Most of these outbreaks were due to a contaminated drinking water supply. The region has a freshwater climate, and clinical cholera cases increase annually between May and October, coinciding with hot summers and monsoons [5].

*V. cholerae* are autochthonous to aquatic environments. During infection, they form biofilm-like aggregates that may play an important role in pathogenesis and disease transmission. Biofilms are also important for the survival of cholera bacteria in the environment [7]. Several previous studies demonstrated that biofilms may cause delayed penetration of antimicrobial agents [8]. With fast-growing multidrug resistance among biofilm-forming *V. cholerae* isolates and a dearth of novel antibiotic research by pharmaceutical industries, there is an urgent need to discover new antibiotic alternatives. Phage therapy can be a potential alternative to antibiotics in the era of multidrug-resistant bacterial infections. The lytic phages, which disrupt bacterial metabolism and lyse the bacteria, are proposed as being useful for phage therapy [9]. Phages have many potential advantages over antibiotics. Phages are less likely to inflict “collateral damage”, or the destruction of gut flora than antibiotics, since they are more host-specific [10]. Phages are common in the environment, and different phages may work together to influence the incidence and distribution of pathogenic *V. cholerae* in aquatic habitats. *Vibrio* phages may also be important in the environmental control of cholera.

We characterized phage VMJ710 isolated in 2015 from a community sewage water sample during an outbreak of cholera in Manimajra, Chandigarh. We carried out genomic characterization and tested the phage for its antibiofilm testing against the MDR *V. cholerae* O1 pathogenic strain. Using an infant mouse model, we tested the preventive effect of the phage to cause diarrhea by giving the phages before the bacterial testing. We also demonstrate the therapeutic potential of this phage.

## 2. Materials and Methods

### 2.1. Sample Collection

We collected sixty-eight sewage water samples from four surveillance sites in Chandigarh between February 2015 and November 2016. Figure S1 shows three sites of sewage sample collection that included hospital sewage, community sewage at Ramdarbar and Raipur Khurd, and three cholera outbreak sites (Manimajra, Ambala, and Ludhiana). The collected water samples were transferred to the enteric laboratory, Postgraduate Institute of Medical Education and Research (PGIMER), at room temperature and processed within 18–24 h after collection.

### 2.2. Bacterial Strains and Growth Conditions Used

Suspected colonies of *V. cholerae* were identified using routine biochemical tests (positive for oxidase, catalase, indole, lysine and ornithine decarboxylase, string test, nitrate reduction, fermentation of mannose and sucrose, negative for arginine dihydrolase and arabinose fermentation). The strains were confirmed via a serotyping kit (Denka Seiken Co., Ltd., Tokyo, Japan). *V. cholerae* O1 biotype El Tor serotype Ogawa VMJ1 strain isolated from the 2015 outbreak of Manimajra, Chandigarh, was used for the isolation and propagation of phage VMJ710. The phage VMJ710 was tested against 26 MDR *V. cholerae* O1 biotype El Tor serotype Ogawa strains (PGIMER culture collection) via the spot assay test on trypticase soy agar (TSA, Becton, Dickinson and Company, Franklin Lakes, NJ, USA) (Tables S1 and S2). *V. cholerae* O1 strains obtained from clinical cases of cholera and sewage water showed acquired non-susceptibility to at least one agent in three or more antimicrobial categories of the following classes of antibiotics: Third-generation cephalosporins, fluoroquinolones, aminoglycosides, tetracyclines, ampicillin, chloramphenicol, and cotrimoxazole were defined as MDR *V. cholerae* [11]. *V. cholerae* serotype O1 (strain ATCC 39315/El Tor Inaba N16961) was used to standardize the induction of cholera in mice. A streptomycin-resistant *V. cholerae* O1 (ELPGI212) strain isolated from a clinical case of cholera during the 2015 outbreak was used for testing antibiofilm activity and efficacy in mouse models.

### 2.3. Phage Isolation

In total, 3 mL of sewage water was mixed with 500 µL of bacterial culture (10^8^ colony forming units (CFU)/mL) and 2 mL of trypticase soy broth (TSB, Becton, Dickinson and Company, Franklin Lakes, NJ, USA). The whole mixture was incubated for 24 h, and the next day, the mixture was centrifuged at 7800× *g* for 15 min. The supernatant was collected and filtered with a 0.45 µm filter (Pall Corporation, Cortland, NY, USA). To assess phage activity (qualitatively) against the host strain, a spot assay followed by a plaque assay was used [11,12]. 

### 2.4. Phage Purification

A single plaque was picked up via a sterile 1 mL micropipette tip, and this single phage was amplified, and all steps were repeated twice [13]. Ten percent of polyethylene glycol 8000 (PEG 8000, Sigma-Aldrich Corporation, Burlington, MA, USA) was added to the phage preparation, and incubated for 20 h at 4 °C. After centrifuging, the mixture at 257,000× *g*, the pellet was resuspended in the SM buffer (10 mM MgSO_4_·6H_2_O). The resuspended mixture was filtered by a 0.45 µm syringe filter. Final purification was carried out using the dialysis method with the help of the dialysis membrane (MWCO 14,000, Himedia Laboratories, Mumbai, India) [14].

### 2.5. Phage Stability

The following two parameters were used to determine phage stability:

#### 2.5.1. pH Stability

For the pH stability test, 200 µL of phage lysate (10^8^ CFU/mL) was added in different tubes containing sterile SM buffer with a range of pH values from 4 to 10. The tubes were incubated at 37 °C for 2 h [15]. The phage titer was determined at a 60 min interval using the double-layer agar plate technique.

#### 2.5.2. Thermal/Temperature Stability

For thermal stability testing, 200 µL of phage lysate (10^8^ plaque-forming units (PFU)/mL) in triplicates was added to the series of 6 tubes and incubated for 2 h at −20 °C, 4 °C, 25 °C, 37 °C, 50 °C, and 60 °C, respectively. The phage titer was tested every 60 min for 2 h using the double-layer agar plate technique [15]. Incubation at −20 °C and 4 °C was achieved in a laboratory freezer and refrigerator, respectively. The water bath was used to maintain the rest of the temperatures. The phage stability at different pH and temperature values was expressed as the phage stability rate (%) [11].

### 2.6. Phage Morphology

Plaque morphology was determined using the plaque assay [14]. The plaque diameter was calculated in millimeters (mm). A drop of the phage suspension was applied to a carbon-coated copper grid and then replaced with a 3% uranyl acetate solution [11]. The grids were examined with the transmission electron microscope, Tecnai G20, at All India Institute of Medical Education and Sciences (AIIMS), New Delhi. The ImageJ software was used to estimate the size of the phage [16].

### 2.7. Genomic DNA Isolation

A phage DNA isolation kit (Norgen Biotek Corporation, Thorold, ON, Canada) was used to extract phage genomic DNA [17]. The phage DNA quality was determined using 1% agarose gel electrophoresis and a Qubit 3.0 fluorometer (Life Technologies, Carlsbad, CA, USA).

### 2.8. Library Preparation, Sequencing, Assembly, and Annotations

A NEBNext Ultra library preparation kit (New England Bioscience, Ipswich, MA, USA) was used to prepare the genomic DNA library, and the Illumina HiSeq 2500 platform was used for the sequencing. The Cutadap tool was used to trim adaptor sequences after processing the FastQ files [18,19]. De novo assembly of the generated reads was executed using an IVA v1.0.8 assembler with default k-mer sizes [20]. GLIMMER 3 and GeneMark tools were used to predict genes from IVA-assembled contigs [21,22]. The genome annotation was performed using the Rapid Annotation Search Tool (RAST v2) [23]. The complete phage genome was scanned with ARAGORN 1.2.36, and CRISPR-CasFinder 4.2.20 to find tRNA and CRISPR-like systems in the phage genome, respectively [24,25]. The circular map of the phage genome was constructed with the CGView tool [26].

### 2.9. Virulence Factors and Antibiotic Resistance Genes

To determine virulence factors and antibiotic resistance genes, in the phage genome, the entire genome was scanned using the VFDB 2019 (Virulence Factor Database) and CARD (Comprehensive Antibiotic Resistance Database) tools, respectively [27,28].

### 2.10. Phylogenetic Tree and Comparative Genomics

The phylogenetic tree was constructed based on multiple sequence alignment using amino acid sequences of the large subunit terminase protein of phage VMJ710 and related *Vibrio* phages with the help of ClustalX and MEGA X. The ViPTree was used to compare the whole genome of the phage VMJ710 with 3234 additional sequenced phage genomes from the virus–host DB (RefSeq release 212) [29,30]. Whole-genome comparison of phage VMJ710 with four closely related *Vibrio* phage genomes, i.e., JSF 14 (KY883639.1), JSF6 (KY883635.1), ICP1_2006 (HQ641351.1, ICP1_2017 (MN419153.1), ICP1_2012) (MH310936.1), was performed using Mauve [31]. To perform core genome analysis, open reading frames (ORFs) were divided into groups based on how many of the four phage genomes were found with respect to phage VMJ710. If an ORF was found in all phages, it was considered part of the core genome; otherwise, it was regarded as part of the accessory genome. The BRIG tool was used to map core and accessory ORFs to the BRIG alignment [32].

### 2.11. Host Range Testing

In total, 26 *V. cholerae* strains were used to assess the phage host range. A lawn of each strain (OD_600_ ~0.6) was made on TSA plates with a sterilized cotton swab. The host range experiment was performed by spotting 20 µL of phage lysate on the bacterial lawns, followed by overnight incubation at 37 °C. A scale system approach was used to identify the zones of clearing, with negative values denoting turbid zones (no lysis) and positive values denoting clear zones [33]. The efficiency of plating (EOP), which was computed by dividing the average PFU of the target bacteria by the average PFU of the host bacteria, was used to measure relative phage killing. Based on EOP values, phages were categorized as highly virulent (0.1 < EOP < 1 ), moderately virulent (0.001 < EOP < 0.099), avirulent (no plaques formed), and reference (EOP = 1).

### 2.12. Time-Kill Assay

To measure the activity (quantitatively) of the phage, a time–kill assay was performed according to a previously described method with some modifications [15]. The bacterial suspension was combined with phage preparation to achieve initial MOIs of 0.01, 0.1, and 1. The control group contained a mixture of bacterial culture and TSB. The experiment was executed at 37 °C using a spectrophotometer (Tecan Group Limited, Mannedorf, Switzerland) with orbital shaking for 4 s. At an interval of 2 h, the OD_600_ values were measured automatically for 20 h.

### 2.13. Antibiofilm Activity of Phage VMJ710 against V. cholerae

The microtiter-plate-based crystal violet assay was used to measure the biofilm capacity of 30 MDR *V. cholerae* O1 isolates [34]. Strains were classified into the following categories: OD ≤ OD_C_ = non-adherent; OD_C_ < OD ≤ (2 × OD_C_) = weakly adherent; (2 × OD_C_) < OD ≤ (4 × OD_C_) = moderately adherent; and (4 × OD_C_) < OD = strongly adherent [35,36]. To test the antibiofilm activity of the phages, 24-hour-old preformed biofilms were grown on the 13 mm polystyrene coverslips and treated at three different phage titers (10^6^, 10^7^, and 10^8^ PFU). After 24 h of incubation, coverslips were washed, vortexed, and placed into an ultrasonic bath for 4 min at 35 kHz frequency to detach the bacterial cells. To enumerate the bacterial count, detached bacterial cells were serially diluted in normal saline (0.85% NaCl) and spread onto TSA plates. For biofilm imaging, biofilms formed on 13 mm coverslips were examined with a scanning electron microscope [11].

### 2.14. Phage Efficacy Testing against V. cholerae O1 Using an Infant BALB/c Mouse Model

#### 2.14.1. Animals and Maintenance

Four-to-five-day-old BALB/c male mice were procured from the animal house, PGIMER Chandigarh, India. The animals were housed in clean polypropylene cages maintained in the animal house with a controlled temperature of (23 ± 2) °C, relative humidity at 50–60%, alternating 12/12 h light/dark cycle, and adequate ventilation. All experimental studies were approved as per guidelines by the institutional animal ethics committee (Ref. No. 93/IEAC/648). The number of animals used in each experiment is depicted in the different legends in the respective figures.

#### 2.14.2. Mouse Cholera Infection Model

Cholera infection was established using the previously described mouse model by Yen et al., with slight modifications [37]. To standardize the minimum infective dose, mice were divided into 4 groups and inoculated with *V. cholerae* O1 El Tor N16961 using a 2 mL syringe fitted with an 18-oral gavage needle. Each of the mice in the group was then inoculated with 50 μL of culture (10^6^, 10^7^, 10^8^ CFU), and the same volume of phosphate-buffered saline (PBS) was given to the negative control group. Mice were sacrificed with the administration of a ketamine/xylazine (K:100 mg/kg + X: 20 mg/kg) cocktail after 24 h, and the small intestines were removed which were mechanically homogenized in PBS buffer (137 mM NaCl, 2.7 mM KCl, 10 mM Na_2_HPO_4_, 1.8 mM KH_2_PO_4_, pH 7.2), and serial dilutions were plated onto Luria Bertani broth (LB broth, Himedia Laboratories, Mumbai, India) media containing 100 µg/mL of streptomycin.

To standardize the incubation period, the minimum infective dose (standardized above) was administered orogastrically. The percentage of mice positive in intestine culture and survival percentage were observed after a specific time interval for each group of mice (4, 6, 8, 10, 12, and 24 h).

#### 2.14.3. Prophylactic and Treatment Efficacy Testing against *V. cholerae*

To estimate whether the phage VMJ710 would be able to survive in the mice intestine, a phage retention study was performed. Mice were dosed with a phage volume of 50 µL (1 × 10^9^ PFU). This experiment was divided into four groups and sacrificed at a specific time interval after phage administration, i.e., at 2, 6, 12, and 24 h. The small intestines were removed and homogenized, as explained in the previous section. The homogenized material was further centrifuged and filtered with a 0.22 µm syringe filter. The quantification of bacteriophages present in the supernatant was performed as explained above in Section 2.3.

**Figure 1:**
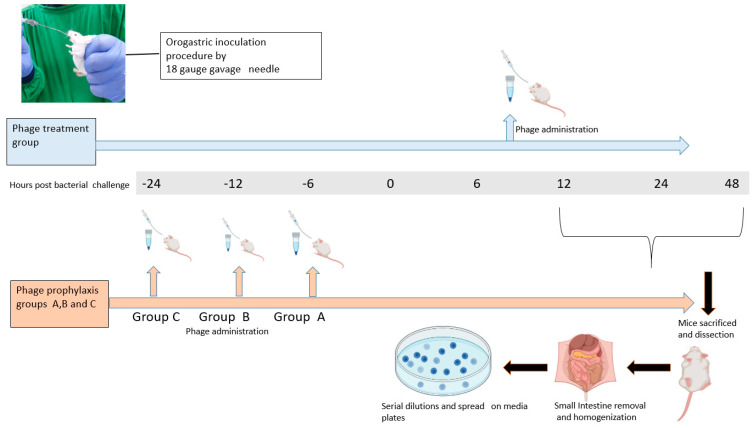
Overview of the procedure used to estimate the efficacy of phage VMJ710 against *V. cholerae* infection using an infant mouse model.

To study the prophylactic efficacy of phage VMJ710, mice were dosed with 50 µL of phage volume (1 × 10^7^ PFU) at 6, 12, and 24 h (three prophylactic groups) before the bacterial challenge (Figure 1). After 48 h of bacterial inoculation, the number of viable *V. cholerae* in the small intestine of sacrificed animals was quantified as described above.

To test the treatment efficacy of *Vibrio* phage VMJ710 against *V. cholerae* strain ELPGI212, animals were divided into control and treatment groups (Figure 1). The treatment group received phage (1 × 10^9^ PFU, at MOI = 1) after 8 h of bacterial challenge. After 12, 24, and 48 h, the number of viable *V. cholerae* in the small intestine of sacrificed animals was quantified as described above. Heamotoxylin and Eosin (H&E) staining was also performed to study the histopathology of the small intestines of mice treated with phage VMJ710.

### 2.15. Nucleotide Sequence Accession Number

The genome sequence of the phage has been submitted to the GenBank database (accession no. MN402506). The raw reads of the phage genome are available at SRA accession number SRR9686322, BioProject accession number PRJNA553871, and BioSample SAMN12253299.

### 2.16. Statistical Analysis

GraphPad Prism 9.0 software was used to run all statistical tests. The time–kill assay, biofilm formation, and phage stability assays were carried out in triplicates. The data were presented as mean ± SD. The Kruskal–Wallis test along with the Dunn post hoc multiple-comparison tests was used to conduct statistical analysis for the time–kill assay and quantitative biofilm assay. A *p*-value of less than 0.05 was considered statistically significant.

## 3. Results

### 3.1. Sample Collection and Phage Isolation

A total of five phages active against *V. cholerae* O1 were isolated from sixty-eight sewage samples. Three *Vibrio* phages, VMJ710, VMJ3, and LDH4, were isolated from the cholera outbreak sites. Only phage VMJ710 (obtained from the community sewage water, Manimajra) could be propagated further.

### 3.2. Phage Morphology

**Figure 2:**
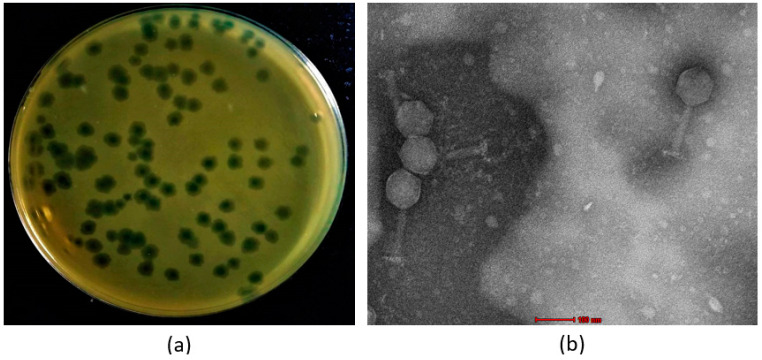
(**a**) Plaque morphology of phage VMJ710. (**b**) Transmission electron microscopy phage of VMJ710 with a scale bar (red) of 100 nm.

Phage VMJ710 produced a clear plaque (4–5 mm in diameter) (Figure 2a). The purified phage particles examined under transmission electron microscopy showed phage VMJ710 to have an icosahedral head of 85 ± 2.4 nm in diameter with a long contractile tail of 130 ± 5 nm, and therefore was classified under the class *Caudoviricetes* (Figure 2b).

### 3.3. Phage Stability

**Figure 3:**
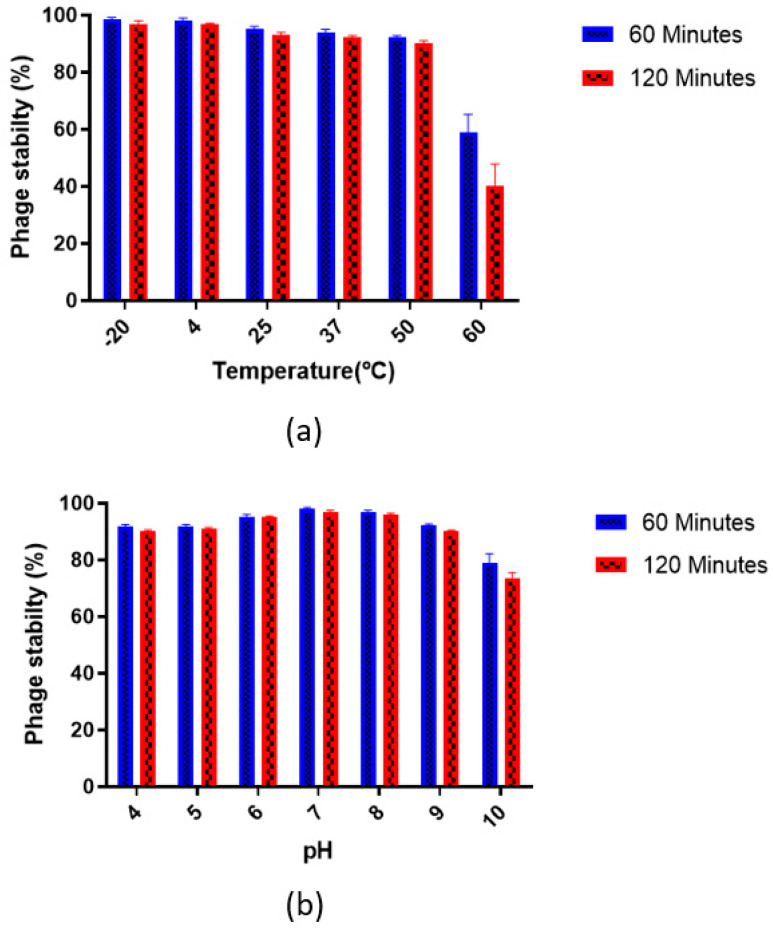
*Vibrio* phage VMJ710 stability rate: (**a**) Thermal stability; (**b**) pH stability. Each data point represents the mean result of experiments performed in triplicates, while the error bars represent the standard deviation.

*Vibrio* phage VMJ710 was highly stable at −20 °C and 4 °C (>95%) after 2 h of incubation. The stability rate was approximately 90% at 50 °C but decreased sharply to <40% at 60 °C (Figure 3a).

The phage was highly stable at pH 7 and pH 8 with a >95% stability rate after 120 min of incubation and was unstable at pH 10 with a stability rate of <75% after 120 min (Figure 3b).

### 3.4. Genome Features of Vibrio Phage VMJ710

**Figure 4:**
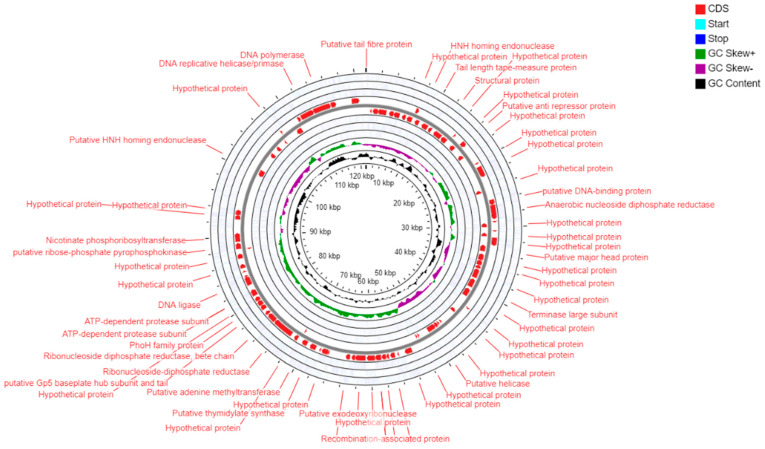
Circular genome map of phage VMJ710.

The complete linear double-stranded DNA genome of phage VMJ710 was 121.4 kb in size, with a GC content of 37.1%. A total of 3,562,707 paired-end raw reads with 150 bp lengths were generated by the Illumina HiSeq sequencer. There were 215 ORFs predicted, and the mean ORF density was 1.76 ORFs per kb. A total of 118 ORFs were present in the direct strand, and 97 ORFs were present in the complementary strand of the phage genome. The putative functions of each ORF are summarized in Table S3. Thirty-five ORFs (17.6%) were predicted to encode functional proteins, whereas 179 ORFs (83.2%) were predicted as hypothetical proteins. Thirty-six functional proteins were classified into different functional groups (Figure 4, Table S3). Out of the thirty-six ORFs, twenty were predicted to encode for DNA replication/metabolism-related proteins such as anaerobic nucleoside diphosphate reductase, HNH homing endonuclease, anaerobic NTP reductase small subunit, putative helicase, recombination-associated protein, putative exodeoxyribonuclease, putative thymidylate synthase, putative adenine methyl transferase, ribonucleotides diphosphate reductase, ribonucleotides diphosphate, reductase beta chain, ATP dependent protease subunit, DNA ligase, putative ribose phosphate pyrophosphokinase, putative antirepressor protein nicotinate phosphoribosyl transferase, DNA replicative helicase/primase, DNA polymerase, and ribonuclease H and PhoH family protein. The ORF 55 was predicted to encode large subunits of terminase involved in phage DNA packaging. It was predicted that seven ORFs encoded structural proteins such as the putative tail fiber protein, putative baseplate component, putative baseplate assembly protein, tail length tape measure protein, and putative major head protein. The ORF 130 was predicted to encode for the host lysis protein putative baseplate hub subunit and tail lysozyme. Furthermore, BLASTP analysis of the VMJ710 genome revealed no similarities to genes encoding for integrase, recombinase, and excisionase. Consequently, the phage VMJ710 was considered a lytic bacteriophage and was selected for further studies. In addition, genome analysis showed that the phage VMJ710 does not contain gene encoding for virulence factors, antibiotic resistance, and CRISPR-Cas.

### 3.5. Phylogenetic Analysis

**Figure 5:**
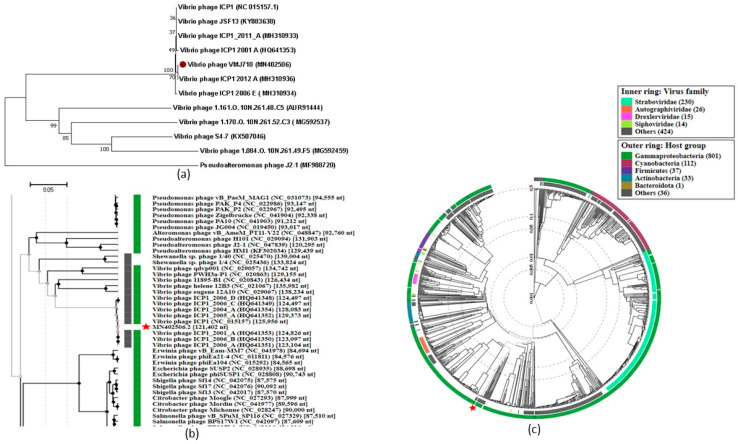
(**a**) Phylogenetic relationship between *Vibrio* phage VMJ710 (red sphere) and the other related phages. (**b**,**c**) are rectangular and circular phylogenetic trees generated using ViPTree [30]. The external and internal rings are colored according to the host bacterial group and virus family, respectively. Red star represents genome of *Vibrio* phage VMJ710.

A phylogenetic tree generated using MEGA X (large terminase subunit based) with other related phages (with an identity of >53.2% in BLASTp) showed that the phage VMJ710 is closely related to the class *Caudoviricetes* phage ICP1_2012_A (Figure 5a). The whole genome-based phylogenetic tree of phage VMJ710 was constructed using Viptree to determine the exact taxonomic position (Figure 5b,c). Phage VMJ710 was placed next to the closely related phages with 121.4–133.6 kb genomes. Phage VMJ710 was classified under the class *Caudoviricetes* in the taxonomical branch *Duplodnaviridae* > *Heunggongvirae* > *Uroviricota* > *Caudoviricetes*.

### 3.6. Comparative Genomics and Core Genome Analysis

**Figure 6:**
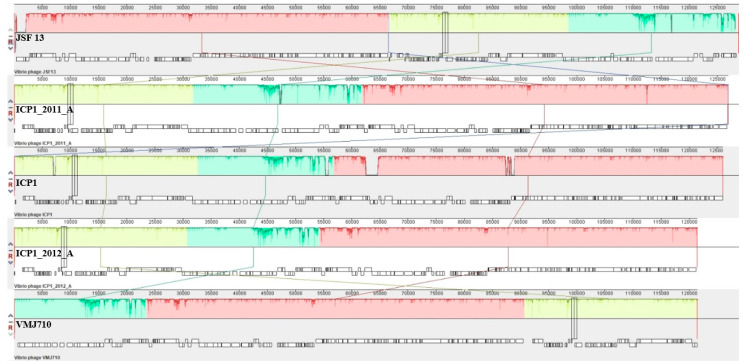
Whole-genome sequence comparison between the genomes of phage VMJ710 and four selected *Vibrio* phages using Mauve 2.0: Annotated CDS (genes) are depicted as white rectangles, with reverse-strand genes, relocated downward. The height of the similarity profile indicates the degree of genomic sequence similarity between the matched regions. Three different colored local collinear blocks (LCBs) illustrate the homologous areas between VMJ710 and four related phages.

**Figure 7:**
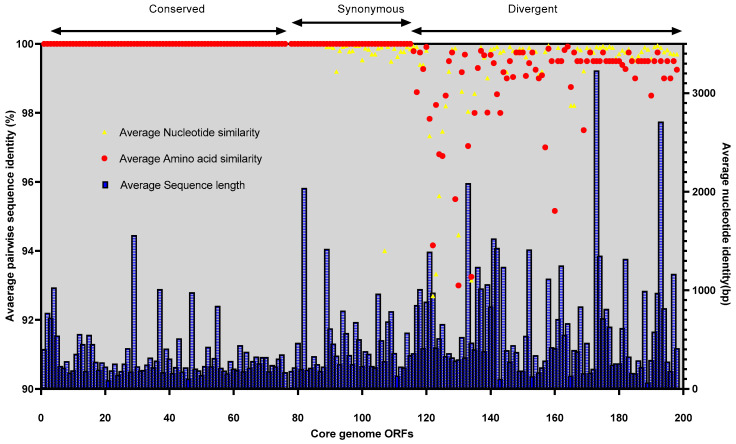
ORF divergence in the core genome. The average pairwise similarity of both DNA nucleotide sequence (yellow triangles and amino acid residue (red dots) alignments are used to organize all ICP1 core-genome ORFs.

The complete genome of the phage VMJ710 was compared with four related phages (Table 1). According to the Progressive Mauve analysis, the genome of each phage stated above has three homologous local collinear blocks (LCBs), and the boundaries of colored blocks represent the breakpoints of genome organization (Figure 6). Mauve revealed a few conserved portions of the aforementioned phages that appear to be devoid of genomic changes internally. Some potential orthologous genes have also been observed and are denoted by the black vertical bars (Figure 6). We observed 92.09% (198/215) core and 7.90% (17/215) accessory ORFs among these five phages. The Brig tool analysis showed that the most variable genome regions of insertions or deletions among the above-mentioned phages encoding for accessory ORFs are shown as gaps (Figure S3). No significant areas of GC content variation were found. Despite being present in all of the genomes included in our investigation, the sequence conservation of the core-genome ORFs varied. The core ORFs were divided into three categories based on average pairwise nucleotide and amino acid sequence identity: conserved-core, synonymous-core, and divergent-core (Figure 7, Table S4).

The conserved core was made up of 87 ORFs that had 100% nucleotide sequence and amino acid sequence identity. The synonymous core includes 27 ORFs with a small amount of nucleotide variability between genomes, but all mutations were silent, resulting in identical amino acid sequences. The remaining 84 ORFs with divergent nucleotide and amino acid pairwise identities made up the divergent core. These divergent ORFs had pairwise identity similarities ranging from 99.9 to 92.7% at the nucleotide level and from 99.95% to 94.16% for amino acid sequences (Table S4).

The accessory ORFs were distributed in three separate groups of patterns A, B, and C throughout all three genomes (Table S5). These ranged from occurrences in three genomes (*n* = 2) to singletons (*n* = 2) with an alternative pattern of accessory ORFs occurrence.

### 3.7. Conserved Functional Domains

The majority of ORFs in the accessory and core genomes were categorized as hypothetical proteins. Only 35 of the core ORFs (17.6%), including 13 in the conserved core, 5 in the synonymous core, and 17 in the divergent core, had a projected function. The accessory core only has one ORF with a putative function (Table S5).

### 3.8. Host Range Testing

Phage VMJ710 was tested against 26 MDR *V. cholerae* isolates and was observed to be active against 12 (46.1%) strains (Figure S2). The highest EOP (>1) was observed against *V. cholerae* 219, 235, ELPGI212, 220, 15-238 isolates, and the lowest EOP (0.001–0.099) was against *V. cholerae* 231, 229, and 221 isolates (Table 2).

**Figure 8:**
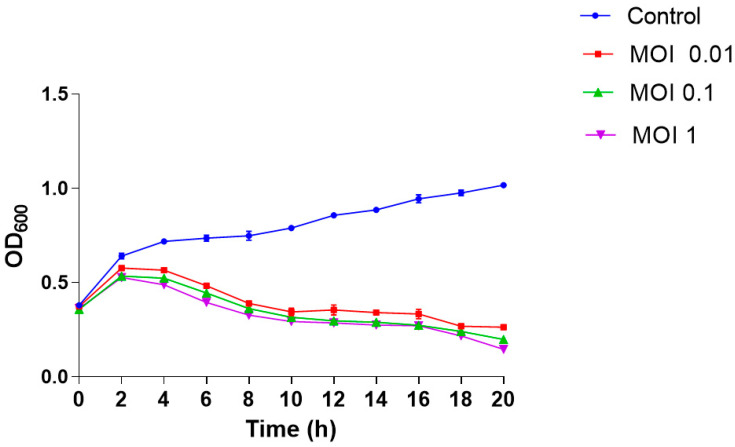
Time–kill assay of phage VMJ710 against MDR *V. cholerae* strains at MOI 1.0, MOI 0.1, and MOI 1. Error bars indicate the standard deviation among triplicate experiments.

The lytic ability of phage was tested against *V. cholerae* ELPGI212 strain at MOIs 0.01, 0.1, and 1.0 using the time–kill assay. No significant reduction in bacterial growth was observed at any MOI up to 2 h of incubation. When compared with the control group, bacterial growth was reduced (*p* < 0.05) after 4–6 h of treatment at all MOIs (Figure 8). Phage VMJ710 was found to be most effective at MOI 1 compared with MOI 0.01 and MOI 0.1.

### 3.9. Anti-Biofilm Activity of Phage VMJ710 against Multidrug-Resistant V. cholerae

**Figure 9:**
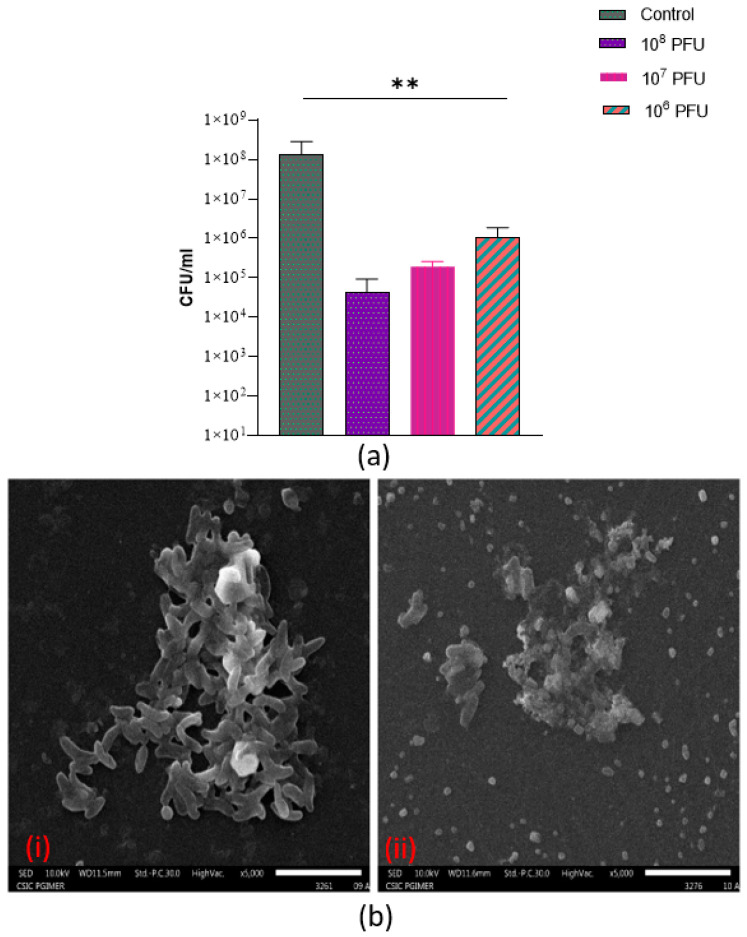
(**a**) Activity of phage VMJ710 at three different titers (10^6^, 10^7^, 10^8^ PFU) on preformed biofilm structure after 24 h incubation in terms of biofilm viable counts. Error bars indicate Mean ± SD. ** indicates a statistically significant difference at the *p*-value < 0.01. (**b**) SEM images at 5000× (magnification) (i) Control group (ii) Effect of phage VMJ710 (10^8^ PFU) on biofilm after 24 h of incubation. Scale bars (white)—5 µm.

The quantitative estimation of biofilm formation of 26 MDR *V. cholerae* strains by the microtiter well test method categorized the strains as follows: non–adherent (7.69%), weak (23.07%), moderate (38.4%), and strong (30.7%) (Figure S4). It demonstrates the production of aggregates and microcolonies, resulting in the formation of a compact and dense biofilm structure covering the surface of the polystyrene coverslip (Figure S5). As 24 h old biofilms were exposed to phages at three different titers (10^6^, 10^7^, and 10^8^ PFU), the biofilm viable counts significantly decreased (*p* < 0.01) (Figure 9a). The phage titer of 10^8^ PFU was found to be most effective to degrade preformed biofilms (Figure 9a, b). The structural architecture of an established 24 h biofilm at 5000× is shown in Figure S5. The SEM results showed bacterial dispersal, clear disruption, and a reduction in the bacterial biofilm structure (Figure 9b).

### 3.10. Efficacy of Vibrio Phage VMJ710 against MDR V. cholerae ELPGI212 in Mice

A bacterial inoculum of 10^7^ CFU (minimum infective dose) after 8 h was found to be the most optimum to induce cholera infection in the infant mice.

**Figure 10:**
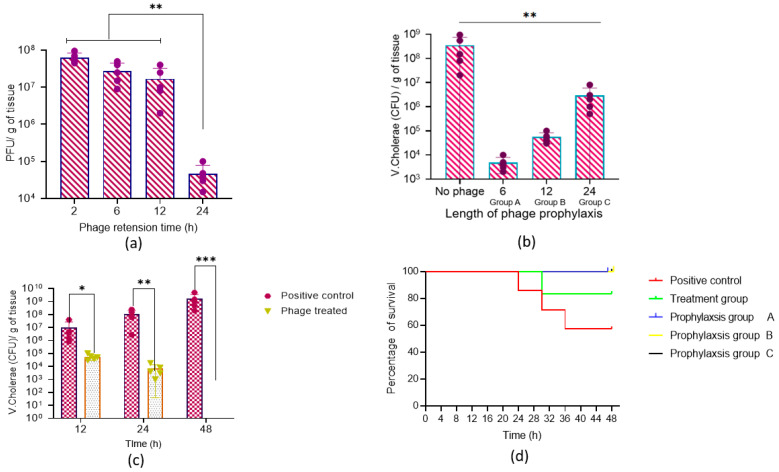
(**a**) *Vibrio* phage number (PFU/g of tissue) retained in the mouse intestine without host bacteria. (**b**) *V. cholerae* (CFU/g of tissue) cells recovered when phages were administered before bacterial challenge as follows: Prophylaxis group A (6 h), prophylaxis group B (12 h), and prophylaxis group C (24 h). (**c**) Bacterial load (CFU/g) recovered from mice intestine tissue after treatment with phage at 12, 24, and 48 h post-infection. (**d**) Percent survival of mice in different groups. Error bars indicate Mean ± SD. The significant difference indicated by *p < 0.05, ** *p* < 0.01, and *** *p* < 0.001.

Before testing the prophylactic effect of the phage to prevent cholera infection, we tested whether phages could be retained in the mice intestine in the absence of host bacteria. When animals were dosed with a high titer (1 × 10^9^ PFU), 1.7 × 10^8^ PFU/g (SD ± 13,769,531) of phages could be recovered after 12 h (Figure 10a). However, at 24 h, a steep decline in the phage titer was observed. The data provided in Figure 10b showed that phage prophylaxis was maximum when mice were dosed with phage VMJ710 before 6 h of bacterial inoculation where the bacterial load was reduced by approximately three orders of magnitude in comparison to the infection control group.

**Figure 11:**
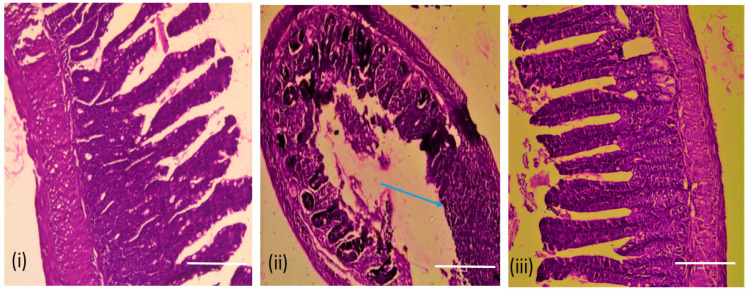
Histopathology of the mouse small intestine: (**i**) Negative control showing normal villus architecture. (**ii**) Infection-only control group; disruption of overlying mucosa and inflammatory cell infiltration (blue arrow). (**iii**) Intestinal architecture after phage treatment at 10^8^ PFU. Scale bar (resolution)-200 µm.

In the treatment group, a reduction in the bacterial count was observed from 1.0 × 10^8^ CFU/g (SD ± 84,660,772) to 7.6 × 10^4^ CFU/g (SD ± 6046) after 24 h, and furthermore, no *V. cholerae* cell was detected after 48 h in comparison to the untreated group, where bacterial counts raised to 1.6 × 10^10^ CFU/g (SD ± 1,737,354,310) (Figure 10c). Animal survival percentage was 83% in the treatment group and 57% in the infection control group (10d). Histopathological analysis of the small intestine of mice infected with *V. cholerae* ELPGI212 showed infiltration of lymphocytes, plasma cells, and disruption of the overlying mucosa, where intestinal microvilli were normal, and no intestinal damage was observed in the phage-treated group. Phage-treated mice showed less damage to intestinal architecture and reduced inflammatory exudate (Figure 11).

## 4. Discussion

Bacteriophages are natural predators of bacteria and are abundantly available. Phages, like all viruses, are very species-specific in terms of their hosts, infecting only a single bacterial species or even distinct strains within a species. Phages are promising alternatives to treat multi-drug-resistant bacteria and the biocontrol of cholera. We isolated *Vibrio* phage VMJ710 from the community sewage water sample in Manimajra, Chandigarh, in 2015 when an outbreak of cholera was occurring in this region. VMJ710 belongs to the class *Caudoviricetes* and is very similar (BLASTn identity 99.96%) to phage ICP1_2012 which was isolated from patients’ stool samples that were collected in India. All-tailed bacterial and archaeal viruses with icosahedral capsids and double-stranded DNA genomes are classified into the class *Caudoviricetes* [38].

Phage VMJ710 has a long contractile tail of 130 ± 5 nm and an icosahedral capsid of 85 ± 2.4 nm length, similar to phage ICP1, which has an 86 nm long icosahedral capsid and a 106 nm long tail [39]. ICP1 phages are dominant phages widespread throughout the Bay of Bengal’s coastline zones [40]. In Bangladesh, during cholera outbreaks, this group of phages is found along with *V. cholerae* in affected patients [40]. The whole-genome sequence of VMJ710 shows the most similarity (BLASTn identity 86–92.97%) to four phages (ICP1_2012_A (MH310936), ICP1 (NC_015157), ICP1_2011_A (MH310933), and JSF13 (KY883638) isolated from different geographic regions across the world in BLASTn analysis. The genome size range of previously studied ICPI (class *Caudoviricetes*) related *Vibrio* phages is 121.4–133.6 kb, similar to VMJ710. Comparative genomics of VMJ710 and related four phages using BRIG and Mauve tools revealed the conservation of nucleotide homology to a larger extent. Phage VMJ710 is closely related to ICP1-like phages of the class *Caudoviricetes,* as demonstrated in the phylogenetic study based on the terminase large sequence and ViPTree. Some of the ICP1 phages possess the CRISPR-Cas (clustered regularly interspersed short palindromic repeats-CRISPR-associated proteins) system, which is involved in bacterial defense against predators such as phages [41]. Another mechanism to resist is the prevention of phage reproduction by hosting phage-inducible chromosomal islands (PICI) [40]. CRISPR-Cas systems are typically found in bacteria and archaea; however, *V. cholerae*- specific ICP1 phages have recently been revealed to contain the CRISPR-Cas system that inactivates PICI-like elements (PLE) in *V. cholerae* [42]. In a previous study, CRISPR-Cas-related sequences were found in five (JSF5, JSF6, JSF13, JSF14, and JSF17) of the 29 sequenced *Vibrio* phages (17.2%) [41]. VMJ710 lacks a CRISPR-Cas sequence, whereas the other two closely related phages (JSF13 and ICP1_2011_A) possess a CRISPR-Cas system. It is presumed that enhanced interactions of phages with cholera bacteria during seasonal epidemics of cholera may be facilitated by the phage-encoded CRISPR-Cas system.

Like previously studied *Vibrio* ICP1 phages, the majority of the phage VMJ710 genome is related to hypothetical proteins, and more than half of ORFs (20/36) with the predicted functions are involved in replication/metabolism-related proteins. A putative Gp5 baseplate hub subunit and a tail lysozyme with N-acetylmuramidase activity were encoded by ORF 132 of phage VMJ710. These components are crucial for locally digesting the peptidoglycan layer to allow the tube to enter the periplasm [43]. ORFs 10 and 11 encode for HNH homing proteins that are involved in endonuclease activity, such as site-specific homing endonucleases [44]. HNH proteins belong to a large Pfam protein family and are associated with nuclease activity in all kingdoms of life [44]. Tailed bacteriophages use terminase enzymes to bundle their enormous double-stranded DNA genomes into a preformed protein shell known as the “prohead”. The ORF 55 was predicted to encode for a large subunit terminase protein which is involved in the translocation of the viral capsid DNA during the final stage of phage assembly. The most prevalent proteins found in the majority of phages in the class *Caudoviricetes*, are terminases [45]. Endonuclease proteins with the HNH motif may interact with phage terminase proteins to facilitate the packaging and maturation of viral DNA [44].

This phage has a host range of 46 % and no bacterial resistance was observed against phage VMJ710 in comparison to a study by Yen et al., who observed bacterial resistance against the ICP1 phage after 4–6 h of treatment [37]. The host range of VMJ710 and ICP1-like phages is limited to *V. cholerae* O1, whereas the host range of ICP2 and ICP3 includes non-O1 *V. cholerae* strains. ICP2 and ICP3 can lyse the *V. cholerae* O139 strain MO10 and the non-O139 strain CR034-24, respectively [39]. Biofilm-forming cells are more resistant to antibacterial agents and may contribute to higher levels of antimicrobial resistance [46,47,48,49] Phages have been considered potential agents to control biofilms [50]. Extracellular polysaccharide material is a well-known component of stable bacterial biofilms and can protect bacteria from desiccation, predation, and bacteriophage attack [51,52,53]. Numerous studies have revealed that the components of the biofilm, its age, the efficacy of the phage, and the length of the treatment play a role in efficient biofilm eradication [54,55]. The findings of our study confirmed the concept that the use of phage can reduce *Vibrio* biofilms, in particular those grown on plastic surfaces. Phage treatment has significantly removed the biofilms grown on polystyrene coverslip surfaces. The SEM results were consistent with the quantitative data, which showed a considerable decrease in biofilm-forming cells and biomass. The phage was stable over a wide range of temperatures and pH when incubated without a host, suggesting that it has good thermal and pH stability. Thus, it would be easy to preserve and beneficial in different environmental conditions in phage therapy.

Our study is unique as we tested both the preventive and treatment potential of phage VMJ710 against a biofilm-forming MDR *V. cholerae* isolate in a mouse model. The infant mouse model is the most commonly used in a majority of cholera studies [56]. Adult mice are not able to colonize efficiently by *V. cholerae* without the elimination of the gut microflora, whereas infant mice are efficiently colonized [57]. Though the exact reason for the colonization of infant mice is not well studied but might be because of their immature or poor immune system. Bacterial counts were reduced significantly, and animals survived within 24–48 h post-bacterial challenge when phages were administered by orogastric inoculation. This indicated that phage preparation needs to be administered within a specified time. The histopathological examination of the intestine of the phage-treated mice revealed lesser tissue damage and almost normal intestinal walls, crypts, and overlying mucosa. This reveals that phage-treated animals had less severe infections and could withstand a deadly bacterial attack.

We also showed that the phage can prevent the establishment of *V. cholerae* infection and prevent disease when the phage was administered 6–12 h before the bacterial challenge. Our results are similar to those of a previous study by Yen et al., where a cocktail of three highly characterized virulent phages (ICP1, ICP2, and ICP3) was similarly tested in infant mice. When phages were administered orally up to 24 h before *V. cholerae* challenge, the colonization of the intestinal tract was reduced, and cholera-like illness was prevented [37]. In our study, though the most significant reduction in bacterial counts occurred at 6–12 h, mouse survival was the same in the 24 h group, suggesting that phages administered orally up to 24 h before *V. cholerae* infection could prevent cholera. This is helpful as a pre-emptive therapy in cholera-outbreak-affected areas. The phage can be given to close contacts of cholera cases, where the secondary attack rate may be as high as 50%. Due to the increasing resistance of *V. cholerae* to antibiotics, alternative therapies are needed. Biocontrol of the disease, as well as the environmental reservoir via phages, can also be a potential alternative to control cholera. Genome analysis provided no evidence of lysogenic genes (obligately lytic), genes related to potential virulence factors, or antibiotic resistance. Based on all these characteristics, phage VM710 is a suitable and promising candidate as a biocontrol and therapeutic agent. However, more such phages need to be discovered for formulating suitable cocktails. Further trials will be needed to ensure the safety of phages for human use.

## 5. Conclusions

We isolated a *Vibrio* phage VMJ710 from the community sewage sample during an outbreak of cholera in Chandigarh, India. WGS revealed that the phage was lytic and devoid of genes associated with lysogeny, virulence, or antibiotic resistance. The phage has anti-biofilm activity and is stable under different environmental conditions. This phage can be a suitable candidate for oral phage administration and potentially combatting cholera infections caused by pathogenic MDR *V. cholerae* strains.

## Figures and Tables

**Table 1:** Properties of *Vibrio* phage VMJ710 and related four *Vibrio* phages.

*Vibrio* Phage Name	Taxonomic Class	Isolation Year	Isolation Source	Genomic Size (bp)	G+C Content	Genbank Accession Number
ICP1	*Caudoviricetes*	2001	Stool	125,956	37.1	HQ641347
ICP1_2012_A	*Caudoviricetes*	2012	Stool	121,418	37.2	MH310936
VMJ710	*Caudoviricetes*	2015	Water	121,402	37.2	MN402506
JSF13	*Caudoviricetes*	2017	Water	128,814	37.2	KY883638
ICP1_2011_A	*Caudoviricetes*	2011	Stool	126,861	37.1	MH310933

**Table 2:** The clinical isolate data including source, date of isolation, antibiotic sensitivity, and killing effect of the phage VMJ710 on the individual bacterial isolates.

Date of Collection, Antibiotic Sensitivity, Source of Isolation, and Phage Killing of *V. cholerae* Strains.														
*V. cholerae* Isolates	Date of Collection	Source	Antibiotic Sensitivity											
			Amikacin	Cefotaxime	Ciprofloxacin	Gentamycin	Norfloxacin	Nalidixic acid	Ampicillin	Furoxan	Chloramphenicol	Tetracycline	Cotrimoxazole	Phage Killing (EOP)
219	10 August 2015	ST												
235	12 August 2015	ST												
66	23 August 2015	ST												
ELPGI212	14 September 2015	ST												
231	14 September 2015	ST												
223	20 September 2015	ST												
220	20 September 2015	ST												
15-238	25 October 2015	ST												
1672	30 October 2015	ST												
VMJ3	8 November 2015	SW												
CHD5	15 November 2015	ST												
LDH4	5 August 2016	SW												
183	19 July 2016	ST												
100	20 August 2016	ST												
221	20 August 2016	ST												
187	11 September 2016	ST												
163	20 September 2015	ST												
174	20 September 2016	ST												
226	16 October 2016	ST												
222	17 October 2016	ST												
238	25 October 2016	ST												
229	25 October 2016	ST												
236	28 October 2016	ST												
218	5 November 2016	ST												
211	10 November 2016	ST												
VMJ1	11 November 2016	ST												
Footnotes ST-Stool SW-Sewage water			Color codes of the antibiotic sensitivity profile							Key to EOP				EOP
														>1.00
														0.100–1.00
					Sensitive									0.001–0.099
					Intermediate									Reference 1.00
					Resistant									No growth

## Data Availability

The data presented in this study are available in this article and data used to support the findings of this study are available from the corresponding author upon request.

## Supplementary Materials

The following supporting information can be downloaded at: https://www.mdpi.com/article/10.3390/antibiotics12061046/s1, Figure S1: Water sample collection sites: 1-Ludhiana, 2-Manimajra, 3-sewage treatment plant, Ramdarbar, 4-main sewage drain, PGIMER, 5-Sewage treatment plant, Raipur Khurd, 6-Ambala. Map was created using the online version of Scribble Maps software. Figure S2: Representative picture of host range determination of *Vibrio* phage VMJ710 by spot assay. Figure S3. Whole-genome sequence comparison between the genomes of phage VMJ710 and four selected *Vibrio* phages using the *BRIG tool* v0.95: Reference genome: phage VMJ710 (Innermost black ring) genome. The phage genome sections that have less than 50% or no resemblance to VMJ710 are shown by a gap in the relevant genome ring. Figure S4: Biofilm formation capacity of 26 MDR *Vibrio cholerae* strains in crystal violet assay. Figure S5: The structural architecture of an established 24-hour-old *V. cholerae* biofilm at 5000×. Table S1: Antibiotic sensitivity pattern of 26 MDR *V. cholerae* strains. Table S2: Host range testing results of *Vibrio* phage VMJ710 against 26 *V. cholerae* strains. Table S3: Functional annotation of *Vibrio* phage VMJ710 genome. Table S4: Details of core ORFs details with an average nucleotide sequence length. Table S5: Accessory-genome ORF occurrence matrix
