# Supplementary material for: Vibrio Phage VMJ710 Can Prevent and Treat Disease Caused by Pathogenic MDR V. cholerae O1 in an Infant Mouse Model
Source: Antibiotics (Basel). 2023 Jun 14;12(6):1046. doi: 10.3390/antibiotics12061046 (PMC10295236; doi:10.3390/antibiotics12061046)
Supplement: Supplementary file 1 [file antibiotics-12-01046-s001.zip › antibiotics-2447508-supplementary.pdf]

Supplementary data

Table S1: Antibiotic sensitivity pattern of 26 MDR *V. cholerae* strains.

Sr No.	*Vibrio* Strain	Amikacin	Cefotaxime	Ciprofloxacin	Gentamycin	Norfloxacin	Nalidixic acid	Ampicillin	Furoxan	Chloramphenicol	Tetracycline	Cotrimoxazole
1	219	I	S	S	R	S	R	S	R	S	S	R
2	235	I	R	S	S	S	R	R	R	S	S	R
3	66	S	S	S	R	S	R	S	R	S	S	R
4	ELPGI212	S	I	R	S	S	R	R	R	S	S	R
5	231	S	S	S	R	S	R	S	R	S	S	R
6	223	S	S	R	S	R	R	S	R	I	S	R
7	220	I	S	S	R	S	R	S	R	S	S	R
8	15-238	S	R	S	S	S	R	S	R	S	S	R
9	1672	S	S	S	R	S	R	S	R	S	R	R
10	VMJ3	S	R	R	S	S	R	R	R	S	I	R

11	CHD5	S	S	S	R	S	R	S	R	R	S	R
12	LDH4	I	S	S	S	R	R	S	R	S	S	R
13	183	S	S	R	S	S	R	S	R	S	S	R
14	100	I	I	R	S	S	R	S	S	R	I	S
15	221	R	I	S	R	S	R	S	R	S	S	R
16	187	S	S	S	S	S	R	R	R	S	S	R
17	163	S	S	R	R	S	R	R	R	S	S	R
18	174	S	S	S	R	S	R	S	R	R	R	R
19	226	S	I	R	S	S	R	S	R	S	S	R
20	222	S	S	S	R	S	R	S	R	S	S	R
21	238	I	S	R	S	S	R	S	R	S	S	R
22	229	S	S	S	R	S	R	S	S	R	S	R
23	236	I	S	S	R	S	R	R	R	R	R	R
24	218	S	S	R	R	R	S	S	S	S	S	R
25	211	R	S	S	R	R	R	S	R	I	R	S
26	VMJ1	I	S	S	S	S	S	R	R	S	R	R

S-Sensitive,R-Resistant,I-Intermediate

Table S2: Host range testing results of *Vibrio* phage VMJ710 against 26 *V. cholerae* strains.

Sr. No.	*V. cholerae* strains	*V. cholerae* Serotype	Phage VMJ710
1	219	Ogawa	+
2	235	Ogawa	+
3	66	Ogawa	+
4	ELPGI212	Ogawa	+
5	231	Ogawa	+
6	223	Ogawa	+
7	220	Ogawa	+
8	15-238	Ogawa	+
9	1672	Ogawa	-
10	VMJ3	Ogawa	-
11	CHD5	Ogawa	-
12	LDH4	Ogawa	-
13	183	Ogawa	-
14	100	Ogawa	-
15	221	Ogawa	+
16	187	Ogawa	-
17	163	Ogawa	-
18	174	Ogawa	-
19	226	Ogawa	-
20	222	Ogawa	-
21	238	Ogawa	+
22	229	Ogawa	+
23	236	Ogawa	-

24	218	Ogawa	-
25	211	Ogawa	-
26	VMJ1	Ogawa	+

(+) effective; (-) not effective

Table S3: Functional annotation of *Vibrio* phage VMJ710 genome.

+ORFs	Start	Stop	Directions	No. of residues	Predicted molecular function
1	607	969	-	120	hp
2	972	1823	-	283	hp
3	1820	3241	-	473	Putative baseplate component
4	3252	3656	-	134	hp
5	3666	4439	-	257	Putative baseplate assembly protein
6	4443	5456	-	337	hp
7	5456	5869	-	137	hp
8	5859	6473	-	204	hp
9	6703	7587	-	294	hp
10	8099	8821	-	240	HNH homing endonuclease
11	9376	10404	-	342	tail length tape-measure protein
12	10635	11177	-	180	hp
13	11234	11695	-	153	hp
14	11697	13088	-	463	Structural protein
15	13220	14197	-	325	hp
16	14197	14616	-	139	hp
17	14622	15137	-	171	hp
18	15239	15919	-	226	hp

19	16098	16826	+	242	Putative antirepressor protein
20	16947	17372	+	141	hp
21	17418	18077	-	219	hp
22	18081	18403	-	110	hp
23	18413	18775	-	120	hp
24	18775	19113	-	112	hp
25	19264	19497	+	77	hp
26	19494	19709	+	71	hp
27	19706	20026	+	106	Acyl carrier protein
28	20153	20371	+	72	hp
29	20371	20559	+	62	hp
30	20516	22183	+	555	hp
31	22189	22608	+	139	hp
32	22674	22961	+	95	hp
33	22966	23265	+	99	hp
34	23268	23549	+	93	hp
35	23551	23802	+	83	hp
36	23932	24459	-	175	hp
37	24629	24793	+	52	hp
38	24828	25016	+	62	hp
39	25099	25419	+	106	hp
40	25431	25787	+	118	hp
41	25800	26594	+	264	Putative DNA binding protein
42	26680	28767	+	695	Anerobic ribunucleoside triphosphate reductase
43	28767	29237	+	156	Anerobic ribunucleoside triphosphate reductase

44	29302	29910	-	202	hp
45	29913	30191	-	92	hp
46	30418	30819	+	133	hp
47	30863	31429	-	188	hp
48	31562	32800	+	412	Putative homing endonuclease
49	32842	33861	-	339	Putative major head protein
50	33887	34270	-	127	hp
51	34286	35347	-	353	hp
52	35356	36192	-	278	hp
53	36202	36759	-	185	hp
54	36864	38390	-	508	Portal protein
55	38619	40049	-	476	Terminase large subunit
56	40303	40404	-	33	hp
57	40401	41639	-	412	hp
58	41710	42102	-	130	hp
59	42117	42392	-	91	hp
60	42541	42984	+	147	hp
61	42981	43436	+	151	hp
62	43480	43854	-	124	hp
63	44188	44370	-	60	hp
64	44372	44920	-	182	hp
65	44931	45386	-	151	hp
66	45400	45744	-	114	hp
67	46756	47007	-	83	hp
68	47028	47213	-	61	hp

69	47213	47395	-	60	hp
70	47398	47673	-	91	hp
71	47676	47870	-	64	hp
72	47873	48103	-	176	hp
73	48100	48639	-	179	hp
74	48636	50051	-	471	Putative helicase
75	50055	50183	-	42	hp
76	50180	50521	-	113	hp
77	50632	50859	-	75	hp
78	50862	51020	-	52	hp
79	51085	51588	-	167	hp
80	51622	51711	-	29	hp
81	51921	52089	-	55	hp
82	52089	52277	-	62	hp
83	52274	52492	-	72	hp
84	52485	52742	-	85	hp
85	52745	52978	-	77	hp
86	52987	53274	+	95	hp
87	53634	54752	+	372	hp
88	54801	54984	+	47	hp
89	55031	55414	+	127	hp
90	55420	55602	+	60	hp
91	55602	55859	+	60	hp
92	55871	56293	+	140	hp
93	56293	57003	+	236	hp

94	57199	57612	+	137	hp
95	57613	58321	+	236	hp
96	58405	59262	+	285	Recombination associated protein rdgc
97	59319	60572	+	417	Putative homing endonuclease
98	60573	60749	+	58	hp
99	60739	62298	+	519	Putative exodeoxyribonuclease
100	62291	62839	+	182	hp
101	62833	63063	+	76	hp
102	63060	63725	+	221	hp
103	63725	63913	+	62	hp
104	63972	64106	+	44	hp
105	64115	64510	+	131	hp
106	64520	64711	+	63	hp
107	64724	64972	+	82	hp
108	64976	65293	+	105	hp
109	65293	65673	+	126	hp
110	65677	65895	+	72	hp
111	65897	66184	+	95	hp
112	66185	66418	+	77	hp
113	66421	67434	+	337	hp
114	67445	67615	+	56	hp
115	67599	67817	+	72	hp
116	67899	68309	+	136	hp
117	68379	69344	+	321	Flavin-dependent thymidylate synthase

118	69375	69683	+	102	hp
119	69673	69834	+	53	hp
120	69834	70670	+	278	Modification methylase FokI
121	70680	70838	+	52	hp
122	70826	71293	+	155	hp
123	71290	71511	+	73	hp
124	71511	72023	+	170	hp
125	72023	72187	+	54	hp
126	72198	72401	+	67	hp
127	72552	75782	+	1076	ribonucleoside-diphosphate reductase
128	75794	77146	+	450	ribonucleoside-diphosphate reductase
129	77252	77746	+	164	hp
130	77743	77919	+	58	hp
131	77931	78149	+	72	hp
132	78146	78862	+	238	Putative baseplate hub subunit and tail lysozyme
133	78893	79705	+	270	hp
134	79717	80349	+	210	hp
135	80419	80601	+	60	hp
136	80611	81591	+	326	ATP dependent clp protease proteolytic subunit
137	81694	81900	+	68	hp
138	81913	82194	+	93	hp
139	82181	82369	+	62	hp
140	82382	83734	+	450	DNA ligase

141	83816	83953	+	45	hp
142	83953	84186	+	77	hp
143	84188	84616	+	142	hp
144	84618	84863	+	81	hp
145	84851	85111	+	86	hp
146	85111	85728	+	205	hp
147	85744	85956	+	70	hp
148	85969	86388	+	139	hp
149	86363	86551	+	62	hp
150	86569	87327	+	252	hp
151	87339	87569	+	76	hp
152	87583	87897	+	104	hp
153	88115	88957	+	280	Putative ribose-phosphate pyrophosphokinase
154	89138	90457	+	439	Nicotinate phosphoribosyltransferase
155	92115	92330	+	71	hp
156	92333	92518	+	61	hp
157	92487	93170	+	227	hp
158	93167	93955	+	262	hp
159	94215	94580	-	121	hp
160	94690	95019	-	109	hp
161	95124	95282	-	52	hp
162	95302	95586	-	94	hp
163	95697	95888	-	63	hp
164	95898	96104	-	68	hp
165	96267	96458	-	63	hp

166	96463	96621	-	52	hp
167	96629	96805	-	58	hp
168	96901	97191	-	96	hp
169	97208	97468	-	86	hp
170	97884	98105	-	73	hp
171	98117	98272	-	51	hp
172	98529	98741	+	70	hp
173	98989	99123	+	44	hp
174	99682	99903	-	73	hp
175	99904	100899	-	331	putative HNH homing endonuclease
176	101491	101670	-	59	Putative homing endonuclease
177	101728	101955	-	75	hp
178	101971	102348	-	125	hp
179	102350	102565	-	71	hp
180	102576	102860	-	94	hp
181	102963	103292	-	109	hp
182	103289	103549	-	86	hp
183	103552	104187	-	211	hp
184	104194	104427	-	87	hp
185	104613	104936	-	107	hp
186	105044	105625	-	193	hp
187	105627	105950	-	107	hp
188	105952	106131	-	59	hp
189	106143	106385	-	80	hp
190	10586	106804	-	72	hp

191	106807	107037	-	76	hp
192	107275	107583	+	102	hp
193	107586	107936	+	116	hp
194	107939	108511	+	190	hp
195	1085081	108678	+	56	hp
196	108685	109026	+	113	hp
197	109045	109227	+	60	hp
198	109382	110356	-	324	Putative primase/helicase
199	110340	110558	-	72	hp
200	110630	111097	+	155	Ribonuclease H
201	111069	111269	+	66	Ribonuclease H
202	111259	113298	+	679	DNA replicative/helicase
203	113314	116025	+	903	DNA directed DNA polymerase
204	116074	116892	+	72	hp
205	116876	117070	+	64	hp
206	117210	117425	+	71	hp
207	117428	117760	+	110	hp
208	117831	118109	+	92	hp
209	118106	118357	+	83	hp
210	118370	118552	+	60	hp
211	118558	118785	+	75	hp
212	118788	118970	+	60	hp
213	119248	120414	+	388	hp
214	120525	120938	-	137	hp
215	120940	121368	-	142	hp

hp-hypothetical protein

Table S4: Details of core ORFs details with an average nucleotide sequence length

	ORF	Average nucleotide similarity (%)	Average Amino acid similarity (%)	Nucleotide standard deviation	Amino acid standard deviation	Average Nucleotide Sequence length
1	ORF4	100	100	0.00	0.00	405
2	ORF5	100	100	0.00	0.00	774
3	ORF10	100	100	0.00	0.00	723
4	ORF11	100	100	0.00	0.00	1029
5	ORF12	100	100	0.00	0.00	543
6	ORF25	100	100	0.00	0.00	234
7	ORF26	100	100	0.00	0.00	216
8	ORF34	100	100	0.00	0.00	282
9	ORF37	100	100	0.00	0.00	165
10	ORF38	100	100	0.00	0.00	189
11	ORF40	100	100	0.00	0.00	357
12	ORF52	100	100	0.00	0.00	558
13	ORF61	100	100	0.00	0.00	456
14	ORF63	100	100	0.00	0.00	183
15	ORF64	100	100	0.00	0.00	549

16	ORF65	100	100	0.00	0.00	456
17	ORF70	100	100	0.00	0.00	276
18	ORF71	100	100	0.00	0.00	195
19	ORF72	100	100	0.00	0.00	271
20	ORF77	100	100	0.00	0.00	228
21	ORF80	100	100	0.00	0.00	90
22	ORF82	100	100	0.00	0.00	189
23	ORF84	100	100	0.00	0.00	258
24	ORF88	100	100	0.00	0.00	144
25	ORF90	100	100	0.00	0.00	183
26	ORF91	100	100	0.00	0.00	258
27	ORF94	100	100	0.00	0.00	414
28	ORF98	100	100	0.00	0.00	177
29	ORF99	100	100	0.00	0.00	1560
30	ORF101	100	100	0.00	0.00	231
31	ORF103	100	100	0.00	0.00	189
32	ORF 106	100	100	0.00	0.00	192
33	ORF107	100	100	0.00	0.00	249
34	ORF108	100	100	0.00	0.00	318
35	ORF110	100	100	0.00	0.00	219
36	ORF111	100	100	0.00	0.00	288
37	ORF113	100	100	0.00	0.00	1014
38	ORF114	100	100	0.00	0.00	171
39	ORF116	100	100	0.00	0.00	411
40	ORF118	100	100	0.00	0.00	309
41	ORF119	100	100	0.00	0.00	162

42	ORF123	100	100	0.00	0.00	222
43	ORF124	100	100	0.00	0.00	513
44	ORF130	100	100	0.00	0.00	177
45	ORF131	100	100	0.00	0.00	219
46	ORF135	100	100	0.00	0.00	103
47	ORF136	100	100	0.00	0.00	981
48	ORF137	100	100	0.00	0.00	207
49	ORF139	100	100	0.00	0.00	189
50	ORF141	100	100	0.00	0.00	141
51	ORF 142	100	100	0.00	0.00	234
52	ORF143	100	100	0.00	0.00	429
53	ORF 151	100	100	0.00	0.00	231
54	ORF152	100	100	0.00	0.00	315
55	ORF153	100	100	0.00	0.00	843
56	ORF155	100	100	0.00	0.00	216
57	ORF156	100	100	0.00	0.00	186
58	ORF161	100	100	0.00	0.00	159
59	ORF162	100	100	0.00	0.00	285
60	ORF164	100	100	0.00	0.00	207
61	ORF165	100	100	0.00	0.00	192
62	ORF167	100	100	0.00	0.00	444
63	ORF176	100	100	0.00	0.00	180
64	ORF178	100	100	0.00	0.00	378
65	ORF179	100	100	0.00	0.00	216
66	ORF180	100	100	0.00	0.00	285
67	ORF181	100	100	0.00	0.00	330

68	ORF182	100	100	0.00	0.00	261
69	ORF185	100	100	0.00	0.00	324
70	ORF187	100	100	0.00	0.00	324
71	ORF188	100	100	0.00	0.00	180
72	ORF189	100	100	0.00	0.00	243
73	ORF191	100	100	0.00	0.00	231
74	ORF192	100	100	0.00	0.00	309
75	ORF193	100	100	0.00	0.00	351
76	ORF195	100	100	0.00	0.00	171
78	ORF197	100	100	0.00	0.00	183
79	ORF199	100	100	0.00	0.00	219
80	ORF200	100	100	0.00	0.00	468
81	ORF201	100	100	0.00	0.00	201
82	ORF202	100	100	0.00	0.00	2040
83	ORF205	100	100	0.00	0.00	195
84	ORF206	100	100	0.00	0.00	216
85	ORF207	100	100	0.00	0.00	333
86	ORF209	100	100	0.00	0.00	252
87	ORF210	100	100	0.00	0.00	183
88	ORF211	100	100	0.00	0.00	228
89	ORF3	99.91	100	0.03	0.00	1422
90	ORF8	99.92	100	0.07	0.00	615
91	ORF13	99.89	100	0.18	0.00	462
92	ORF24	99.2	100	0.12	0.00	339
93	ORF35	99.8	100	0.17	0.00	252
94	ORF41	99.92	100	0.06	0.00	795

95	ORF47	99.95	100	0.07	0.00	567
96	ORF66	99.78	100	0.12	0.00	345
97	ORF67	99.8	100	0.17	0.00	252
98	ORF78	99.95	100	0.07	0.00	679
99	ORF79	99.95	100	0.07	0.00	504
100	ORF85	99.53	100	0.05	0.00	234
101	ORF89	99.87	100	0.15	0.00	384
102	ORF96	99.84	100	0.05	0.00	358
103	ORF112	99.7	100	0.16	0.00	234
104	ORF115	99.7	100	0.19	0.00	219
105	ORF117	99.87	100	0.10	0.00	966
106	ORF129	99.9	100	0.15	0.00	495
107	ORF138	94	100	0.16	0.00	282
108	ORF157	99.92	100	0.07	0.00	684
109	ORF158	99.49	100	0.34	0.00	789
110	ORF159	99.86	100	0.13	0.00	366
111	ORF173	99.63	100	0.29	0.00	135
112	ORF177	99.78	100	0.17	0.00	228
113	ORF190	99.77	100	0.18	0.00	219
114	ORF194	99.91	100	0.09	0.00	573
115	ORF196	99.85	100	0.13	0.00	342
116	ORF1	99.93	99.79	0.15	0.32	363
117	ORF2	99.91	98.6	0.07	0.16	852
118	ORF6	99.4	99.75	0.52	0.40	1014
119	ORF7	99.39	99.27	0.34	0.69	414
120	ORF9	99.81	99.91	0.05	0.19	885

121	ORF14	97.33	97.83	2.71	2.10	1392
122	ORF15	92.7	94.16	6.32	2.10	978
123	ORF16	93.33	98.23	3.85	1.92	420
124	ORF17	95.59	96.8	1.41	1.81	516
125	ORF21	97.46	96.75	2.10	2.41	660
126	ORF22	98.2	98.5	2.10	2.31	333
127	ORF23	99.2	99.5	1.21	1.32	363
128	ORF27	99.84	99.75	0.13	0.15	321
129	ORF32	99.87	95.5	2.71	3.81	288
130	ORF33	94.46	93	3.22	4.10	300
131	ORF36	98.62	99.18	1.70	0.62	528
132	ORF39	99.69	99.69	0.219	0.43	321
133	ORF42	98.04	97.04	1.17	0.43	2088
134	ORF43	93.15	93.25	3.97	3.89	471
135	ORF46	98.56	98	1.92	2.31	402
136	ORF48	99.3	99.3	0.10	0.43	1239
137	ORF49	99.9	99.8	2.4	0.49	1020
138	ORF50	99.61	99.67	0.21	0.77	384
139	ORF51	99.01	98.01	0.37	0.11	1062
140	ORF52	99.83	99.68	0.45	0.40	837
141	ORF54	99.86	99.44	0.06	0.10	1527
142	ORF55	99.91	98.54	0.08	2.12	1431
143	ORF56	99.75	98	2.53	3.41	102
144	ORF57	99.8	99.18	0.06	0.32	1239
145	ORF58	99.18	99	0.10	0.43	393
146	ORF59	99.91	99.5	0.15	0.43	276

147	ORF60	99.8	99.04	0.09	0.43	444
148	ORF62	99.73	99.75	0	0.08	375
149	ORF68	99.86	99.75	0.233	0.43	186
150	ORF69	99.86	99.75	0.233	0.43	186
151	ORF73	99.62	99.07	0.245	0.61	540
152	ORF74	99.89	99.44	0.100	0.45	1416
153	ORF75	99.75	99.75	0.43	0.43	129
154	ORF76	99.78	99.25	0.12	0.43	342
155	ORF81	99.1	99	0.52	0.86	168
156	ORF83	99.2	99.09	0.18	1.21	219
157	ORF86	99.77	97	4.5	2.50	288
158	ORF87	99.95	99.86	0.04	0.116	1119
159	ORF92	99.5	99.5	0.113	0.43	424
160	ORF93	95.16	95.16	0.09	4.21	413
161	ORF95	99.89	99.5	0.09	0.41	711
162	ORF97	99.5	99.5	0.12	4.21	1253
163	ORF100	99.82	99.82	0	0.50	549
164	ORF102	99.92	99.92	0.06	0.50	666
165	ORF104	98.21	98.75	1.31	2.61	135
166	ORF105	98.21	99.75	0.45	0.45	396
167	ORF109	99.87	99.5	0.11	0.50	381
168	ORF120	99.82	99.5	0.14	0.43	837
169	ORF121	99.21	97.5	0.54	0.42	159
170	ORF122	99.89	99.5	0.17	0.50	468
171	ORF125	99.84	99.75	2.4	0.52	165
172	ORF126	99.75	99.5	0.21	0.48	204

173	ORF127	99.91	99.5	0.09	0.50	3231
174	ORF128	99.89	99.5	0.07	0.43	1353
175	ORF132	99.93	99.75	0.10	0.50	717
176	ORF133	99.87	99.5	0.15	0.50	813
177	ORF134	99.92	99.5	0.14	0.40	633
178	ORF140	99.89	99.5	0.12	0.50	246
179	ORF144	99.59	99.5	0.24	0.54	261
180	ORF145	99.71	99.5	0.16	0.52	261
181	ORF146	99.81	99.39	0.23	0.63	618
182	ORF154	99.75	99.27	0.19	0.23	1320
183	ORF160	99.85	99.75	0.18	0.50	330
184	ORF163	99.61	99.5	0.23	0.65	163
185	ORF166	99.52	99	0.29	1	159
186	ORF168	99.65	99.5	0.25	0.51	291
187	ORF174	99.77	99.5	0.21	0.52	222
188	ORF175	99.9	99.5	0.09	0.50	996
189	ORF183	99.84	99.5	0.23	0.51	66
190	ORF184	99.57	98.5	0.29	0.64	294
191	ORF186	99.91	99.5	0.08	0.45	582
192	ORF198	99.95	99.75	0.07	0.46	975
193	ORF203	99.89	99.5	0.09	0.51	2712
194	ORF204	99.76	99	0.18	0.49	819
195	ORF208	99.82	99.5	0.21	0.50	279
196	ORF212	99.72	99	0.23	1.10	183
197	ORF213	99.7	99.5	0.19	0.49	1167
198	ORF214	99.7	99.25	0.21	0.08	414

Table S5: Accessory-genome ORF occurrence matrix

Occurrence	ORF name	ICP1	ICP1_2012_A	JSF13	ICP1_2011_A				
	ORF 44					A			
	ORF 170					A			
	ORF 18					B			
	ORF 19					B			
	ORF 20					B			
	ORF 28					B			
	ORF 29					B			
	ORF 30					B			
	ORF 31					B			
	ORF 147					B			
	ORF 148					B			
	ORF 149					B			
	OR150					B			
	ORF 169					B			

	ORF 171					B			
	ORF 45					C			
	ORF 172					C			

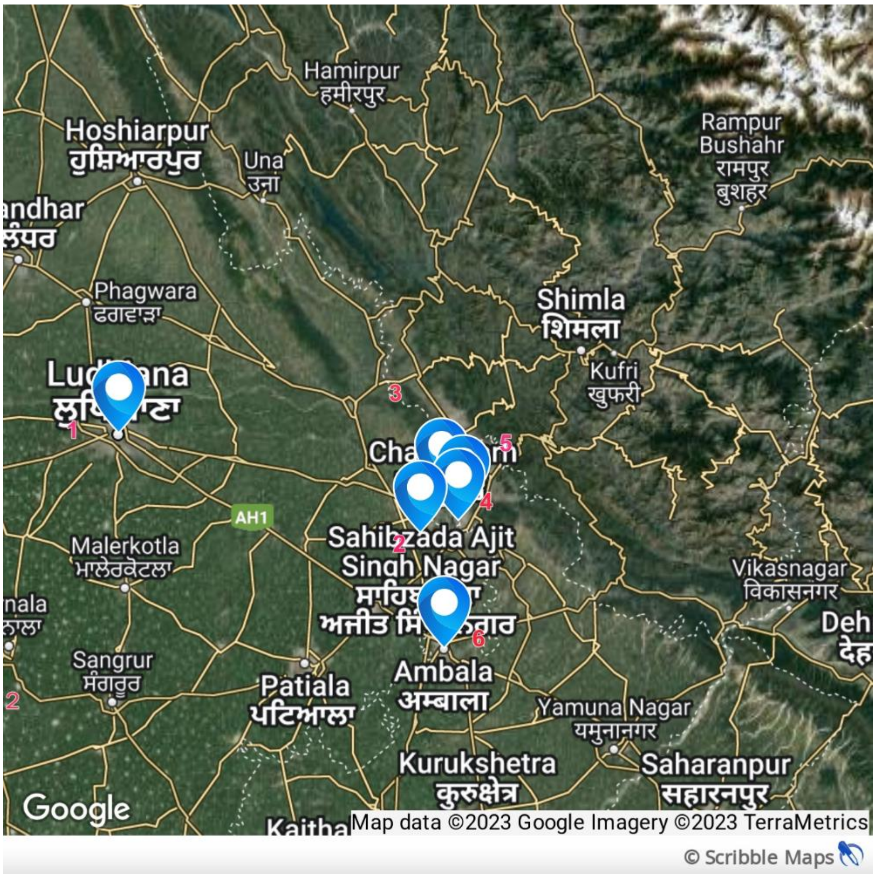

Figure S1: Water sample collection sites:1-Ludhiana,2- Manimajra,3- sewage treatment plant, Ramdarbar,4- main sewage drain, PGIMER,5- Sewage treatment plant, Raipur Khurd,6- Ambala. Map was created using the online version of Scribble Maps software.

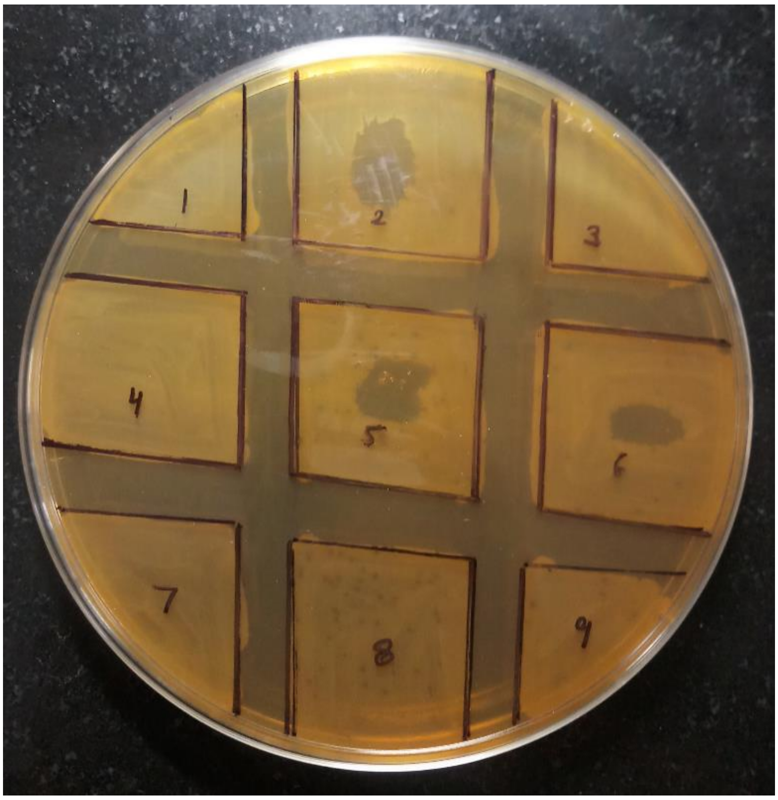

Figure S2: Representative picture of host range determination of *Vibrio* phage

VMJ710 by spot assay.

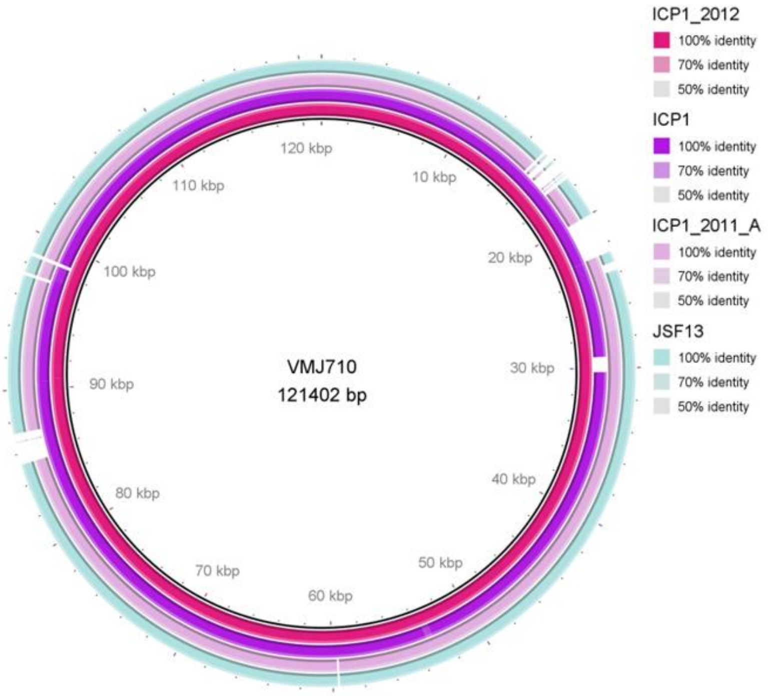

Figure S3. Whole-genome sequence comparison between the genomes of phage VMJ710 and four selected *Vibrio* phages using the *BRIG tool* v0.95: Reference genome: phage VMJ710

(Innermost black ring) genome. The phage genome sections that have less than 50% or no resemblance to VMJ710 are shown by a gap in the relevant genome ring.

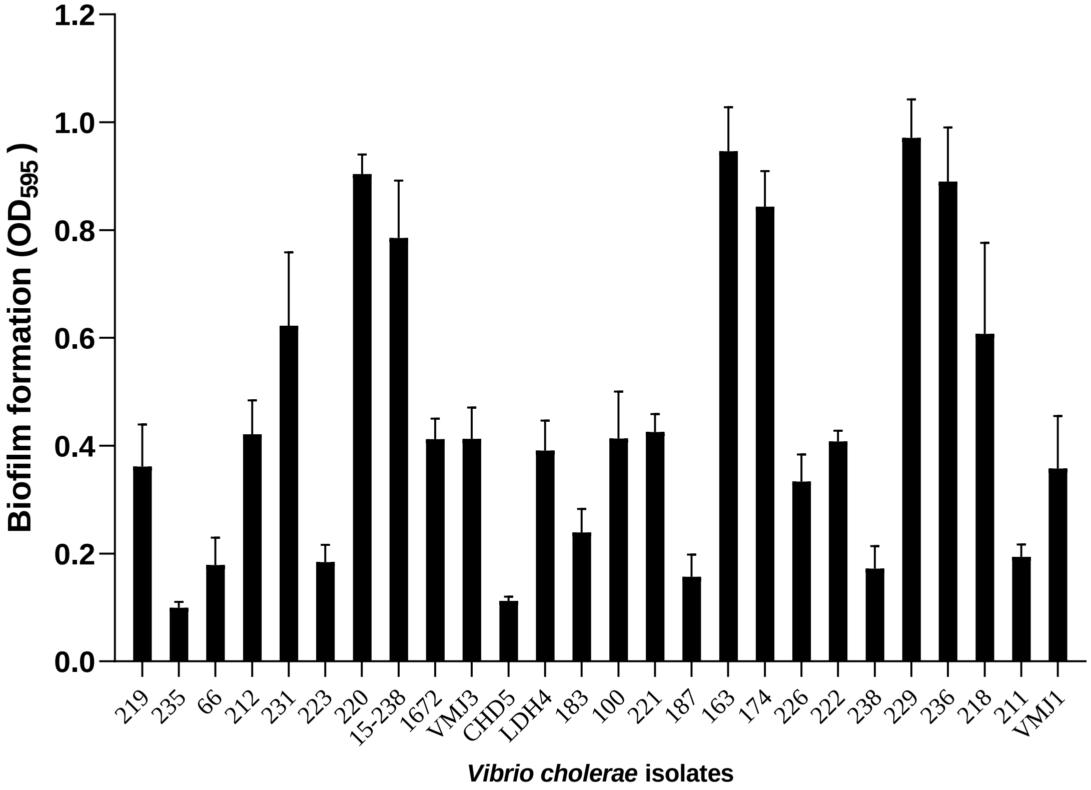

Figure S4: Biofilm formation capacity of 26 MDR *Vibrio cholerae* strains in crystal violet assay.

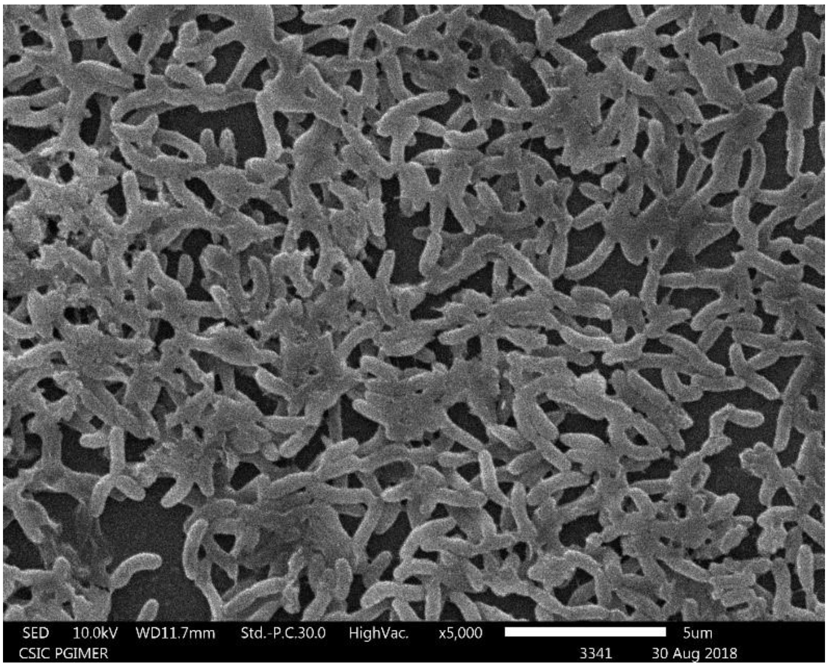

Figure S5: The structural architecture of an established 24 hrs old *V. cholerae* biofilm at 5000 X.
